# Faculty appointment and promotion in Taiwan’s medical schools, a systematic analysis

**DOI:** 10.1186/s12909-022-03435-2

**Published:** 2022-05-10

**Authors:** Jiunn-Tyng Yeh, Boaz Shulruf, Hsin-Chen Lee, Pin-Hsiang Huang, Wen-Hua Kuo, Tyzh-Chang Hwang, Chen-Huan Chen

**Affiliations:** 1 Department of Medicine, Yang Ming Campus, National Yang Ming Chiao Tung University College of Medicine, 155 Li-Long St., Sec. 2, Shih-Pai, Taipei, 112 Taiwan R.O.C.; 2 Office of Medical Education, University of New South Wales, Sydney, Australia; 3 Institute of Pharmacology, National Yang Ming Chiao Tung University College of Medicine, Taipei, Taiwan; 4 Department of Medicine, Taipei Veterans General Hospital, Taipei, Taiwan; 5 Institute of Science, Technology and Society, National Yang Ming Chiao Tung University, Taipei, Taiwan; 6 Institute of Public Health, National Yang Ming Chiao Tung University College of Medicine, Taipei, Taiwan; 7 Department of Medical Education, Taipei Veterans General Hospital, Taipei, Taiwan

**Keywords:** Journal impact factor, Research, Faculty, Medical education

## Abstract

**Background:**

A rigorous faculty appointment and promotion (FAP) system is vital for the success of any academic institution. However, studies examining the FAP system in Asian universities are lacking. We surveyed the FAP policies of Taiwan’s medical schools and identified an overreliance on the CJA score (manuscript Category, Journal quality, and Author order). The potential shortcomings of this metric and recommendations for refinement were discussed.

**Methods:**

We obtained the FAP documents from all 12 medical schools in Taiwan, and analyzed their use of traditional versus non-traditional criteria for FAP according to a published methodology. The influence of the journal impact factor (JIF) on the FAP process was quantified by comparing its relative weight between papers with two extreme JIFs. To better understand the research impact and international standing of each school, we utilized the public bibliographic database to rank universities by the number of papers, and the proportions of papers within the top 10% or 50% citation.

**Results:**

Compared with other countries, Taiwan’s medical schools focus more on the quantifiable quality of the research, mostly using a “CJA” score that integrates the category, JIF or ranking, and authorship of a paper, with the JIF being the most influential factor. The CJA score for an article with a JIF of 20 can be up to three times the threshold for promotion to Assistant Professor. The emphasis on JIF is based on a presumed correlation between JIF and citation counts. However, our analysis shows that Taiwan’s medical schools have lower-than-average citation counts despite a competitive rank in the number of publications.

**Conclusions:**

The JIF plays an unrivaled role in determining the outcome of FAP in Taiwan’s medical schools, mostly via the CJA system. The questionable effectiveness of the current system in elevating the international standing of Taiwan’s higher-education institutions calls for a re-examination of the FAP system. We recommend a reduction in the relative importance of CJA score in the FAP system, adopting more rigorous metrics such as the h-index for evaluating research quality, and supporting more research aimed at improving the FAP system.

**Supplementary Information:**

The online version contains supplementary material available at 10.1186/s12909-022-03435-2.

## Background

A robust and effective faculty appointment and promotion (FAP) system is crucial for promoting scholars' general welfare and nourishing a healthy academic culture in any academic institution. This includes medical schools, which demand even more prudent considerations in light of an extraordinary stake to society. With the rapidly changing academic landscape, such as the emergence of open-access journals, preprint server and the detrimental effect of the COVID-19 pandemic, many stakeholders including academics themselves have started scrutinizing the validity of the traditional approaches for FAP [1]. For example, an expert panel had been convened to discuss the caveats of the traditional criteria for FAP and recommended new ones such as rewarding open publishing culture, promoting responsible research, and funding research on research metrics or evaluation itself [2]. However, a cross-sectional study of the FAP documents in biomedical research institutions worldwide showed that the “non-traditional” criteria such as open access, citation-based metrics, or adherence to publishing guidelines are seldom considered [3]. The majority of the institutions surveyed are still utilizing simple, easily-quantifiable metrics such as the journal impact factor (JIF) or the number of publications as an indicator for research output [3–5].

Several studies have systematically analyzed the FAP documents from universities in western countries and critically examined their utilization of different criteria and metrics [2, 3, 5, 6]. However, the FAP system in Asian medical schools has not been examined in a similar manner. Taiwan, an island nation with a population of around 24 million, is known for its high-quality healthcare system that ranked the third in Bloomberg’s health efficiency ranking [7, 8]. One pillar of Taiwan’s success is its competent public health and clinical personnel trained mainly by the 12 undergraduate medical schools and their respective affiliated institutions [9]. Despite these remarkable records, these institutions have also been marred by academic frauds resulting in article retractions and indignations [10–13]. It is not clear whether the risk-taking behavior in committing academic fraudulence may bear some relationship with the extra incentivization of publishing high-profile articles—a policy almost universally endorsed in Taiwan’s medical institutions.

To appraise the academic environment of medical institutions in Taiwan, we obtained the FAP documented from all 12 medical schools and analyzed the adaption of traditional and non-traditional research evaluation criteria based on the methodology developed in an international cross-sectional study [3]. Using this approach permitted us to directly compare the situation in Taiwan with the previously published cohort. Furthermore, we quantitatively investigated the impact of objective metrics (e.g., the JIF) on the FAP. Using data from the Leiden ranking, an international ranking system on the scientific impact, we also compared research outcomes between these domestic medical schools and some reputable medical institutions in different continents. The aims for this study were 1) to systematically examine the FAP system in Taiwan, and 2) to provide potential strategies for the improvement of the country-specific system in the global context.

## Methods

### Collection of the FAP documents in Taiwan’s medical schools

In Taiwan, most medical education programs offer a 6-year training for students right after they graduate from high school. There is only one institution with a post-baccalaureate program, which was not included in this analysis. The official FAP policies and guidelines for existing faculty members and appointment of new faculty were obtained from the medical schools’ websites (full and abbreviated names of the medical schools, and the websites for the documents are listed in Supplement sheet 1). The documents were all written in Chinese, which were analyzed by authors who are native speakers.

### Analysis of traditional and non-traditional research evaluation criteria

The traditional and non-traditional criteria were searched in the FAP documents based on the methodology developed in Rice et al. (Table 1). We cross-referenced the institutions sampled in Rice et al. and found no overlap with the medical schools in our study. The original FAP documents were screened for any statement concerning the required criteria for promotion to Assistant Professor (Supplement sheet 2), Associate Professor (Supplement sheet 3), or Full Professor (Supplement sheet 4).

**Table 1:** The presence of traditional and non-traditional FAP criteria in Taiwan’s medical schools compared to an international cohort. The numbers are percentages

	Assistant Professor		Associate Professor		Full Professor	
Criteria	**Taiwan (*N* = 12)**	Rice cohort (*N* = 49)	**Taiwan (*N* = 12)**	Rice cohort (*N* = 79)	**Taiwan (*N* = 12)**	Rice cohort (*N* = 26)
**Traditional criteria**						
1. Any quantitative or qualitative mention about publications required	**100**	80	**100**	96	**100**	95
2. Any quantitative or qualitative mention about the specific authorship order in publications	**100**	22	**100**	35	**100**	34
3. Any mention of journal impact factor	**100**	24	**100**	30	**100**	28
4. Any mention of grant funding	**0**	53	**67**	63	**67**	67
5. Any mention requiring that research is recognized at a national or international level	**0**	22	**17**	33	**17**	47
**Non-traditional criteria**						
6. Any mention of citations	**0**	24	**0**	29	**0**	28
7. Any mention of data sharing	**0**	2	**0**	1	**0**	1
8. Any mention of publishing in open access mediums	**0**	0	**0**	0	**0**	0
9. Any mention of registration (including preregistration challenge) of studies	**0**	0	**0**	0	**0**	0
10. Any mention made of adherence to reporting guidelines for publications	**0**	0	**0**	0	**0**	0
11. Any mention of alternative metrics for sharing research (e.g., social media and print media)	**0**	6	**0**	4	**0**	2
12. Any mention of accommodations or adjustments to expectations due to employment leave (e.g., parental leave, medical leave)	**33**	45	**33**	35	**33**	35

### Quantitative analysis of the citation metrics’ influence on FAP

**Fig. 1:**
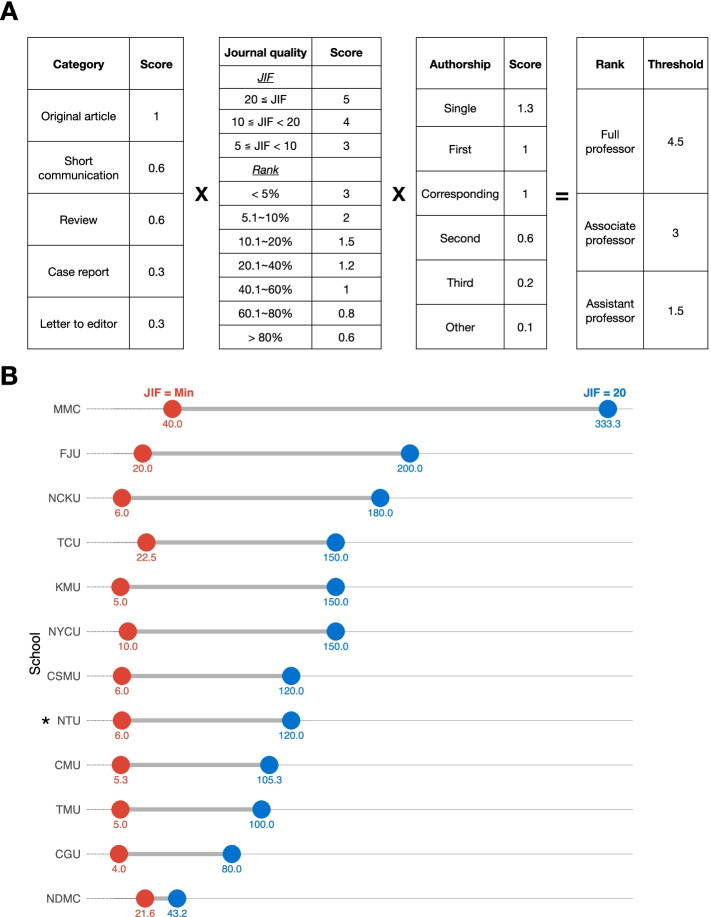
**A** Formula of the CJA system using MMC as an example. **B** Relative weights of JIF on the promotion to Assistant Professor in the medical schools in Taiwan. The number below each blue or red circle marks the weighted score (in percentage) calculated by dividing the score of an original, first-authored manuscript with a JIF of 20 (blue circles) or a lowest-ranked JIF (red circles) by the minimal requirement for a promotion. See Supplement sheet 1 for the full name of the medical schools. *Of note, the influence of the JIF for NTU was calculated from the annual faculty evaluation process instead of the FAP policy, please see more details in the Results section

With only one exception (see Results), a quantitative “CJA” score was utilized to calculate the minimal requirement for FAP in every medical school analyzed. The CJA scores consider three aspects for a published manuscript: the Category (C; e.g., review, original article, case report, etc.), the Journal "quality" (J; e.g., the JIF or ranking of the journal), and the order of Authorship (A). The final score of a publication is calculated by multiplying each score in these three aspects (see example in Fig. 1A). While some differences may exist among these 12 medical schools, the general rules are the same: Original research articles score higher than other types of manuscripts; first and corresponding authorships share a similar score which is higher than that of other co-authors; for the journal's quality assessment, they universally use JIF or SCI/SSCI journal ranking for SCI/SSCI-indexed papers. Since the SCI/SSCI journal ranking is based on JIF in each research domain, domain-specific journal ranking is usually not very different from the one based on JIF except that the former ranking does take into consideration the differences in the size of fields.

Since the weights of articles’ category and authorship are similar across all institutions, we focused our analysis only on the impact of the scores based on the journal quality (i.e., JIF). We calculated the CJA scores for two extreme, make-believe conditions: a first-author original article with either a JIF of 20 or the lowest possible journal ranking. Then these scores were divided by the minimal requirement for promotion to an Assistant Professor. Take Mackay Medical College (MMC) for example (Fig. 1A): The score for first-authorship is 1, and that for the original full-length article is 1; the score for a JIF of 20 is 5, but 0.6 for the article of the lowest rank. Hence the CJA score for a JIF = 20 article is 5, and for a lowest-ranked article is 0.6. The minimal requirement of the CJA score at MMC for promotion to an Assistant Professor in basic science departments is 1.5, so the weights for an article with a JIF of 20 is 5 / 1.5 = 333%, but 0.6/1.5 = 40% for the lowest-ranked article (Fig. 1B).

### Assessing the scientific impact using the Leiden ranking

**Fig. 2:**
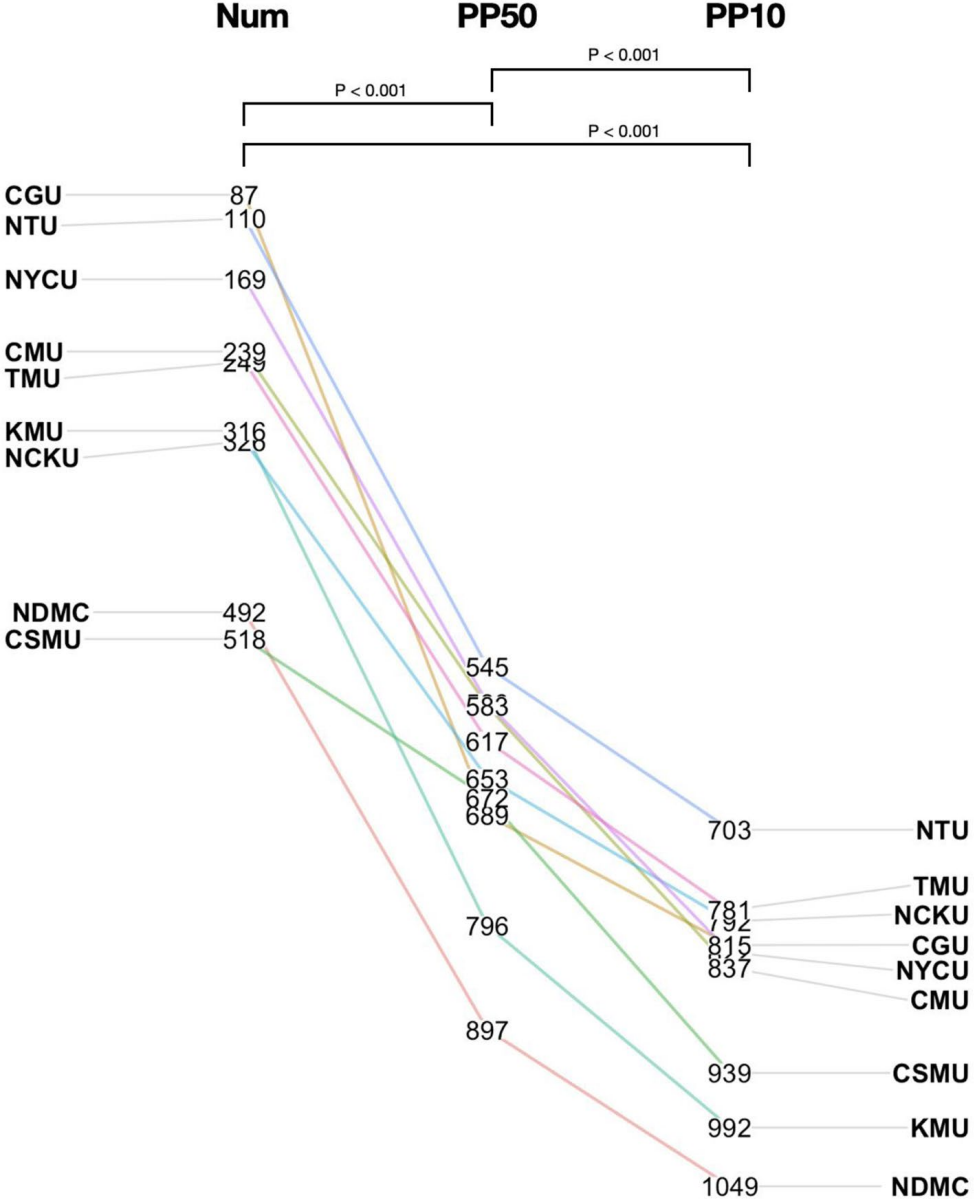
Leiden ranks of medical schools in Taiwan. Numbers represent the rankings. See Methods for definition of Num, PP10, PP50 rankings. See Supplement sheet 1 for the full name of the medical schools

**Fig. 3:**
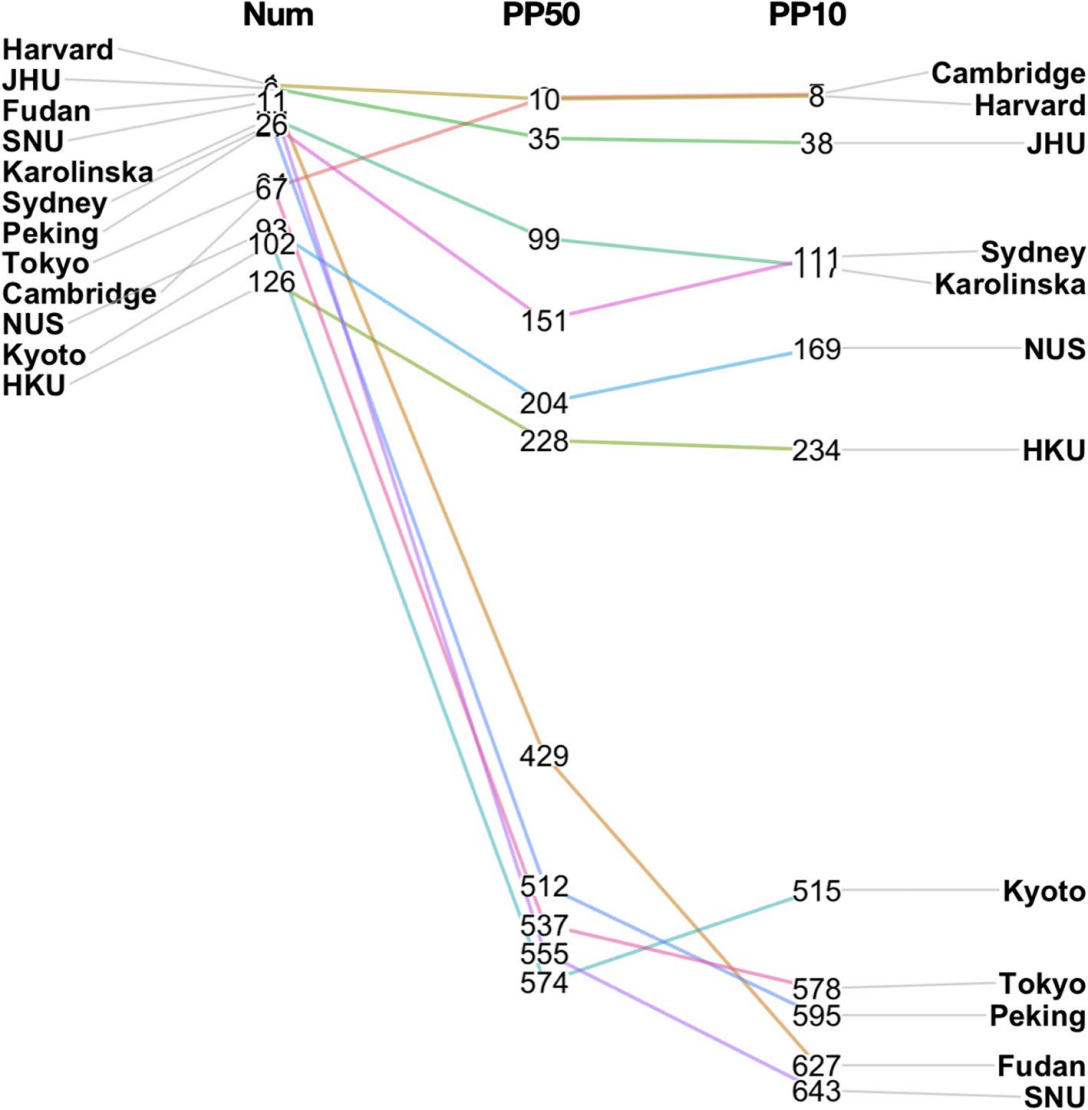
Leiden ranks of medical schools in prestigious medical schools across the globe. Numbers represent the rankings. See Methods for definition of Num, PP10, PP50 rankings. Harvard: Harvard University (U.S.), JHU: Johns Hopkins University (U.S.), Fudan: Fudan University (China), SNU: Seoul National University (South Korea), Karolinska: Karolinska Institute (Sweden), Sydney: University of Sydney (Australia), Peking: Peking University (China), Tokyo: University of Tokyo (Japan), Cambridge: University of Cambridge (U.K.), NUS: National University of Singapore (Singapore), Kyoto: Kyoto University (Japan), HKU: University of Hong Kong (Hong Kong)

To evaluate the relative global research impact of Taiwan’s medical schools, we gathered the 2020 Leiden ranking in biomedical and health science for each institution (https://www.leidenranking.com/ranking/2020/list). The Leiden ranking for scientific impact gathers the bibliometrics data from the Web of Science database and calculates the citation received for research papers published by each institution. A total of 1,071 institutions across the world were included in the 2020 Leiden ranking. According to its website, only universities that have produced at least 800 Web of Science indexed publications in the period 2015–2018 were listed in the 2020 Leiden ranking; hence three medical schools (Mackay Medical College, Fu Jen Catholic University, Tzu-Chi Medical University) were not included in the list of our analysis as they did not reach this threshold (Fig. 2). Results based on three different ways of ranking were obtained and visualized in Fig. 2: The label “Num” is the rank of the institution by the number of publications; the “PP50” the rank by the proportion of publications of an institution belonging to the top 50% in citation counts in their respective field; the “PP10” the proportion of publications within the top 10% in the field. To make a more global comparison, in Fig. 3 we also gathered these ranks for several renowned medical schools on different continents.

### Statistical analysis

Multiple paired Student’s *t*-tests via the R program (Version 4.1.0) were conducted for the comparisons in Fig. 2. *P*-values smaller than 0.05 were deemed statistically significant.

## Results

Our analysis of every medical school’s FAP policy in Taiwan showed a similar procedure in the process of evaluation for academic hiring or promotion. The candidates are assessed in three domains: service (or administrative work), research, and teaching. Each school has its own guideline that gives different weightings to these three attributes based on the results of past peer evaluations and the feedback from their students. Depending on the level of the professorial position, the FAP system imposes different minimum requirements for each area being considered. A complete dossier includes a standardized form with supporting documents attached. The dossier is then sent to reviewers outside the institution. Upon completion of external review, the candidate’s accomplishments are then discussed and voted at three levels of intramural committee: department, school and university. For a new recruit to Assistant Professor, usually only the research aspect is reviewed; otherwise, the FAP system is similar for the new recruits and internal faculty applying for promotion.

As the scoring criteria for service and teaching are highly variable among these medical schools, the current study focuses on the review criteria for evaluating research performance. For cross-country comparisons, we adopted the experimental design in a cross-sectional cohort study by Rice et al. (will be referred to as the Rice cohort hereinafter) [3]. The Rice cohort sampled 170 institutions from Leiden ranking of world universities in the field of “Biomedical and Health Science” and evaluated their utilization of traditional criteria, which emphasize more the quantity of research, as well as the non-traditional criteria, which underscore the quality or reproducibility of research for the FAP process. As shown in Table 1, for the traditional criteria, the medical schools in Taiwan rely more heavily on criteria 1 to 3 compared the Rice cohort. This is because of the adoption of the CJA system, which sets thresholds for an integrated score that is calculated from the multiplication of the category (C), journal impact factor or ranking (J) and authorship (A). Only one school (National Taiwan University, NTU) recently cancelled utilizing the CJA system for the appointment and promotion. The replacement has a similar concept to the CJA system which requires applicants to publish first- or corresponding-authored papers in high-ranking journals (See Supplement sheet 2–4). Also, the CJA system is still implemented by NTU during the annual faculty evaluation, which prevents the faculties from filing a promotion request if they fail the evaluation. Compared to the Rice cohort, the FAP system in Taiwan’s medical schools also places less emphasis on their investigators’ national or international recognitions (Table 1, Criterion 5).

For the non-traditional criteria regarding data sharing, open access, study registration, reporting guideline adherence and altimetric (Criteria 6 to 11), the adoption rate is low if not zero for medical schools in Taiwan. Moreover, evaluations based on actual number of citation or citation-based metrics (Criterion 6) were not implemented in Taiwan. Thus, when assessing research performance, the integrated scoring system (i.e., the CJA system) based on the quantity, authorship, and impact factors is ubiquitously employed and constitutes the most prevalent evaluation tool in Taiwan’s medical schools.

Figure 1A shows an example of the CJA formula used by Mackay Medical College (MMC). In its CJA system, the only factor evaluating the quality of research is the JIF or journal ranking. As described in Methods, we quantified the relative weights of the CJA score on the minimum requirement for promotion between two fictional first-authors with two extremes of the JIF (Fig. 1B). A paper with a JIF of 20 will be counted up to 300% of the minimal requirement for a promotion to Assistant Professor at MMC; whereas that of a low JIF weighs only 4% at CGU (Fig. 1B). Of note, as mentioned above, the NTU recently removed the CJA from the FAP system but still kept it in the annual faculty evaluation, whose outcome directly influences the eligibility for promotion. Hence, we still calculate the influence of the JIF during the faculty evaluation process in NTU. JIF significantly impacts the weight of the CJA scores on the decision made by the FAP system, although its final impact may vary among these medical schools. In summary, in Taiwan's medical schools, the JIF serves as the most important, if not the only, factor determining the perceived research quality of a scientist, and a high-JIF article could bear an overwhelming power over other considerations in the FAP process.

The heavy reliance on the JIF for research evaluation in the FAP system in Taiwan was based on the premise that as JIF represents the average citation of a journal, the higher the average citation a journal receives, the more “impactful” to the scientific community the journal is. Hence when an investigator publishes in high-JIF journals, he/she is likely to contribute more significantly to the field because of the presumed more frequent citations of his/her work. To directly examine the relationship between this perceived impact and the actual citation rate in a global context, we compared the results of three different ranking metrics for 9 domestic medical schools from the 2020 Leiden ranking in biomedical and health science (Fig. 2): the ranking of number of publications (Num), the proportion of publications within the top 50% most-cited ones in the field (PP50), and the proportion of publications within the top 10% most-cited ones (PP10). Among the 1,071 institutions listed in the 2020 Leiden ranking in biomedical and health science, all the medical schools in Taiwan included in the list are among the top 50% when judged by the numbers of publications (Num). The top three Taiwanese medical schools were placed in positions similar to reputable universities in other Asian countries (e.g., Singapore, South Korea, or Japan; Fig. 3). However, the ranks based on PP50 and PP10 are significantly lower (*p*-values < 0.05 by multiple paired Student’s t-test) for all 9 medical school assessed (Fig. 2). This observation suggests that although the total output of research in Taiwan’s medical schools is noteworthy, the overall visibility and impact are lagging behind.

## Discussion

This nationwide Taiwanese study is the first published study that systematically examines the FAP policy for medical schools worldwide. Compared to those presented in the Rice cohort (an international sample of biomedical institutions), the research evaluation process in Taiwan ubiquitously adopts a strictly quantitative approach using a CJA system (Table 1). In this CJA system, the JIF or journal ranking is the most influential factor, and the only factor judging the quality of the research. As the analysis in Fig. 1 shows, the CJA score one earns by publishing a first-author original study in a journal with a JIF of 20 can be up to three times the threshold for an Assistant Professor hiring. The rationale of using JIF as the indicator of research quality and impact is based on the assumption that the JIF is a good proxy for the actual citation of the study. However, despite the heavy incentivization of publishing in high-JIF journals, our analysis shows that the actual numbers of citation for articles published by researchers in Taiwan’s medical schools trail behind those by their counterparts in the west (and some in the east).

The observed discrepancy between the research output and the actual citation is perhaps not surprising since the JIF has been shown to correlate poorly with a given article’s number of citations partly because the distribution of the number of citations for articles published in a journal is highly skewed; thus, the JIF, an arithmetic average of citations for all articles, simply cannot represent the number of citations for any single article [14–16]. If the JIF of an article fails to signify how often the article actually gets cited, one has to ask whether the FAP system implemented is serving its essential role: selecting high-quality academics to be promoted in medical schools or more generally in any science and technology oriented higher-education institutions.

Some may argue that high-JIF journals (e.g., *Nature, Science, Cell, NEJM*) become authorities in the scientific community because of their strict peer-review systems and high rejection rates [17, 18]. Research suggested that the studies published in the highest-JIF journals do not necessarily have better quality, as publishing in high JIF journals is so incentivized in every aspect of the academia that this "JIF frenzy" may have inadvertently jeopardized the quality of the research in the high-ranked journals [19, 20]. Institutions in several countries, including Taiwan, have provided monetary rewards for publishing in high-JIF journals [21, 22]. This degree of emphasis and incentivization on the JIF may have undesirable consequences. Studies have demonstrated higher prevalence of fraud and retraction and potentially lower methodological stringency across basic and clinical research fields [19, 23–34]. World-renowned scientists and institutions have raised serious concerns over using JIF to evaluate investigators’ research performance and proposed for a major overhaul. In the past decade, a swath of manifestos decrying JIF’s prominence and offering alternatives have been promulgated by governments [35, 36], large international organizations or meetings [37, 38], and scientific journals such as *PLOS*, *eLife,* and *Nature* [39, 40].

It is worth noting that a similar discrepancy between the number of publications and the actual citation exists in some otherwise prestigious Asian medical schools, such as the University of Tokyo, Japan and Peking University, China (Fig. 3). However, for those with stronger historical connections with the western education systems, such as the National University of Singapore and the Hong Kong University, the gaps seem smaller. Hopefully the current study will prompt similar investigations across Asia and other non-western higher education systems and medical schools to provide further insights into other factors that may influence research impact on medical institutions globally.

The unremitting evolution of a publish-friendly environment includes the emergence of preprint servers, open access journals, and other novel publishing models. In this light, institutions that want to attract and retain the brightest minds should acknowledge the deficits of JIF and reform their FAP systems. This is both necessary and readily workable, as proposed by an expert panel [2]. In short, they concluded that researchers should be recognized for addressing societal needs and advancing an honorable research culture; their research should be assessed based on validated and responsible indicators, and scientists should be rewarded when the studies are published with transparency regardless of the results. With these aims in mind, the h-index and its derivatives have been developed and are now the most often recommended and utilized metrics for evaluating research achievement on the individual basis, as well as for research groups, institutions, and countries [41–44]. An *h-index* is derived as “A scientist has index *h* if *h* of his/her *N*_*p*_ paper have at least *h* citations each and the other (*Np* – *h*) papers have ≤ *h* citations each”[41].

Specifically designed for evaluating scientists, the h-index bears two distinct advantages. First, it combines the number of publications (an index for productivity) and the number of citations (a proxy for quality) into a single number that can be easily calculated from the citation database [45]. Second, unlike the JIF, the h-index is insensitive to the few highly-cited articles [46]. It is also insensitive to a large set of less-cited papers [47]. Thus, h-index offers an assessment of a scientist’s long-term, overall productivity and quality in research output. However, the h-index is dependent on the field of the research and the length of the career [41, 48]. Although, in some circumstances, the h-index needs to be standardized or adjusted based on the field or the scientists' career length, using the h-index for the FAP process—particularly in Taiwan—is relatively convenient since the applicants for new jobs or for promotions generally are in the similar field of research and have a comparable length of career. We recommend replacing the JIF with the h-index in the CJA system, and introduce more factors or ways of evaluation into the FAP system, such as peer-review or validated structural questionnaires to reduce the dominance of the CJA system.

The JIF-based FAP system in Taiwan’s medical schools was established prior to the internet era 30 years ago; it is debatable whether this system bears the flexibility and capacity to meet the challenges of expedient scientific advancement in the twenty-first century. While medical communities all over the world have taken an evidence-based approach for patient care, it seems appropriate that academic institutions such as medical schools should adopt a similar approach to hire and promote their teaching/research workforces. Considering the fact that both research and research on research are evolving at a remarkable speed, we strongly encourage funding of studies designed to develop more optimal ways to assess the quality of science and scientists should be encouraged. We believe studies regarding the faculty cultivating process in Asian countries are important for the diversity of the field of medical education.

The strength of this study is that all the medical schools in Taiwan are included in the analysis, which provides a comprehensive overview with less bias. Also, the adoption of the published methodology (i.e., the Rice cohort) and public ranking database (i.e., the Leiden Ranking) enables a fair international comparison. The major limitation of this study is that although we had shown a definite over-reliance on JIF during the FAP process and provided and evidence against using the JIF for research evaluation, it is difficult if not possible to demonstrate the causality between the predominance of JIF and the research outcome. Prospective studies or even a randomized controlled trial may be able to provide stronger evidence. Another limitation is that this study focuses mainly on the research evaluation for the faculty appointment and promotion. The criteria and evaluation processes for other aspects such as teaching and service are less clear and highly varied, making it difficult to compare or analyze the effectiveness of the system. Take the evaluation of teaching performance for example, the NDMC evaluates its faculty using a combination from peer-, student- and self-evaluation using self-devised questionnaires; on the other hand, NCKU’s evaluation is based on a list of factors with different weights including course hours, hours for faculty development courses, whether the faculty is the main instructor, student’s response, and other honors associate with teaching. The factors adopted and processes for summarizing the performance are different between medical schools, making it very difficult to compare using a standardized framework. Future studies that take teaching and service into consideration are warranted.

As discussed above, the FAP system employed influences the publishing behavior of existing and future medical faculty. By crafting a system in which the merit of every researcher is judged by a numeric value based on JIF, all medical schools in Taiwan are now in an arms race that may not serve the best interests of the whole scientific community or the society. We therefore reckon that it is perhaps timely for an overhaul: design a FAP system that values the real impact of a paper and the genuine accomplishments of academics in medical schools and beyond.

## Conclusion

From our systematic examination of the FAP policies from every medical school in Taiwan, we found that the JIF plays an unrivaled role in determining the outcome evaluation and promotion of the faculty, mostly via the CJA system. However, based on the international ranking the effectiveness of the current system is questionable and deviates from international trend. Our findings serve as an alarm for the international medical education community regarding the appointment of faculties, as well as a call-to-action for a re-examination of FAP policy for Taiwan’s higher-education institutions. We recommend replacing the JIF with more rigorous metrics (e.g., h-index and its derivatives) for research quality. Prospective researches should also be supported to examine the efficacy of the system reform.

## Supplementary Information

**Additional file 1. **

## Data Availability

The datasets used and/or analyzed during the current study are available from the corresponding author on reasonable request.

## Abbreviations

FAP: Faculty appointment and promotion
JIF: The journal impact factor

## References

[1] Schimanski LA, Alperin JP: The evaluation of scholarship in academic promotion and tenure processes: Past, present, and future. F1000Res 2018, 7:1605. 10.12688/f1000research.16493.1 PMC6325612 30647909

[2] Moher D, Naudet F, Cristea IA, Miedema F, Ioannidis JPA, Goodman SN (2018). Assessing scientists for hiring, promotion, and tenure. PLoS Biol.

[3] Rice DB, Raffoul H, Ioannidis JPA, Moher D (2020). Academic criteria for promotion and tenure in biomedical sciences faculties: cross sectional analysis of international sample of universities. BMJ.

[4] Seglen PO (1997). Why the impact factor of journals should not be used for evaluating research. BMJ.

[5] McKiernan EC, Schimanski LA, Munoz Nieves C, Matthias L, Niles MT, Alperin JP: Use of the Journal Impact Factor in academic review, promotion, and tenure evaluations. Elife 2019, 8. 10.7554/eLife.47338 PMC6668985 31364991

[6] Alperin JP, Munoz Nieves C, Schimanski LA, Fischman GE, Niles MT, McKiernan EC: How significant are the public dimensions of faculty work in review, promotion and tenure documents? Elife 2019, 8. 10.7554/eLife.42254 PMC6391063 30747708

[7] Asia Trounces U.S. in Health-Efficiency Index Amid Pandemic [https://www.bloomberg.com/news/articles/2020-12-18/asia-trounces-u-s-in-health-efficiency-index-amid-pandemic]

[8] Wang CJ, Ng CY, Brook RH (2020). Response to COVID-19 in Taiwan: Big Data Analytics, New Technology, and Proactive Testing. JAMA.

[9] Chou JY, Chiu CH, Lai E, Tsai D, Tzeng CR (2012). Medical education in Taiwan. Med Teach.

[10] Cha S-T, Tan C-T, Chang C-C, Chu C-Y, Lee W-J, Lin B-Z, Lin M-T, Kuo M-L (2017). Retraction Note: G9a/RelB regulates self-renewal and function of colon-cancer-initiating cells by silencing Let-7b and activating the K-RAS/β-catenin pathway. Nat Cell Biol.

[11] Shyu K-G, Wang B-W, Chang H (2016). Retraction: Hyperbaric oxygen activates discoidin domain receptor 2 via tumour necrosis factor-α and the p38 MAPK pathway to increase vascular smooth muscle cell migration through matrix metalloproteinase 2. Clin Sci.

[12] Retraction. The FASEB Journal 2018, 32(4):2316–2316. 10.1096/fj.201700932RRRET 29592790

[13] Notice of Retraction (2016). Arteriosclerosis. Thrombosis, and Vascular Biology.

[14] Zhang L, Rousseau R, Sivertsen G (2017). Science deserves to be judged by its contents, not by its wrapping: Revisiting Seglen's work on journal impact and research evaluation. PLoS One.

[15] Seglen PO (1994). Causal relationship between article citedness and journal impact. J Am Soc Inform Sci.

[16] Kiesslich T, Beyreis M, Zimmermann G, Traweger A (2021). Citation inequality and the Journal Impact Factor: median, mean, (does it) matter?. Scientometrics.

[17] Pudovkin AI. Comments on the Use of the Journal Impact Factor for Assessing the Research Contributions of Individual Authors. Front Res Metr Anal. 2018;3. https://www.frontiersin.org/article/10.3389/frma.2018.0000210.3389/frma.2018.00002

[18] Kurtz MJ, Henneken EA (2017). Measuring metrics - a 40-year longitudinal cross-validation of citations, downloads, and peer review in astrophysics. J Am Soc Inf Sci.

[19] Casadevall A, Fang FC (2014). Causes for the persistence of impact factor mania. mBio.

[20] Brembs B (2018). Prestigious Science Journals Struggle to Reach Even Average Reliability. Front Hum Neurosci.

[21] Cash bonuses for peer-reviewed papers go global [https://www.sciencemag.org/news/2017/08/cash-bonuses-peer-reviewed-papers-go-global]

[22] Quan W, Chen B, Shu F (2017). Publish or impoverish: An investigation of the monetary reward system of science in China (1999–2016). Aslib J Inf Manag.

[23] Fang FC, Casadevall A (2011). Retracted science and the retraction index. Infect Immun.

[24] Fang FC, Steen RG, Casadevall A (2012). Misconduct accounts for the majority of retracted scientific publications. Proc Natl Acad Sci U S A.

[25] Steen RG (2011). Retractions in the scientific literature: do authors deliberately commit research fraud?. J Med Ethics.

[26] Brembs B, Button K, Munafo M (2013). Deep impact: unintended consequences of journal rank. Front Hum Neurosci.

[27] Szucs D, Ioannidis JP (2017). Empirical assessment of published effect sizes and power in the recent cognitive neuroscience and psychology literature. PLoS Biol.

[28] Obremskey WT, Pappas N, Attallah-Wasif E, Tornetta P, Bhandari M (2005). Level of evidence in orthopaedic journals. J Bone Joint Surg Am.

[29] Lau SL, Samman N (2007). Levels of evidence and journal impact factor in oral and maxillofacial surgery. Int J Oral Maxillofac Surg.

[30] Bain CR, Myles PS (2005). Relationship between journal impact factor and levels of evidence in anaesthesia. Anaesth Intensive Care.

[31] Tressoldi PE, Giofre D, Sella F, Cumming G (2013). High impact = high statistical standards? Not necessarily so. PLoS One.

[32] Pandis N, Fleming PS, Worthington H, Salanti G (2015). The Quality of the Evidence According to GRADE Is Predominantly Low or Very Low in Oral Health Systematic Reviews. PLoS ONE.

[33] Chess LE, Gagnier J (2013). Risk of bias of randomized controlled trials published in orthopaedic journals. BMC Med Res Methodol.

[34] Saginur M, Fergusson D, Zhang T, Yeates K, Ramsay T, Wells G, Moher D (2020). Journal impact factor, trial effect size, and methodological quality appear scantly related: a systematic review and meta-analysis. Syst Rev.

[35] The metric tide: Report of the independent review of the role of metrics in research assessment and management [ blogs.lse.ac.uk/impactofsocialsciences/files/2015/07/2015_metrictide.pdf]

[36] Evaluation of Research Careers fully acknowledging Open Science practices [https://ec.europa.eu/research/openscience/pdf/os_rewards_wgreport.pd]

[37] San Francisco Declaration on Research Assessment [https://sfdora.org/read/]

[38] Hicks D, Wouters P, Waltman L, de Rijcke S, Rafols I (2015). Bibliometrics: The Leiden Manifesto for research metrics. Nature.

[39] Schekman R, Patterson M (2013). Reform Res Assess Elife.

[40] Time to remodel the journal impact factor (2016). Nature.

[41] Hirsch JE (2005). An index to quantify an individual's scientific research output. Proc Natl Acad Sci U S A.

[42] van Raan AFJ (2006). Comparison of the Hirsch-index with standard bibliometric indicators and with peer judgment for 147 chemistry research groups. Scientometrics.

[43] Kinney AL (2007). National scientific facilities and their science impact on nonbiomedical research. Proc Natl Acad Sci U S A.

[44] Csajbók E, Berhidi A, Vasas L, Schubert A (2007). Hirsch-index for countries based on Essential Science Indicators data. Scientometrics.

[45] Hirsch JE (2007). Does the H index have predictive power?. Proc Natl Acad Sci U S A.

[46] Liu Y, Ravichandra Rao IK, Rousseau R (2009). Empirical series of journal h-indices: The JCR category Horticulture as a case study. Scientometrics.

[47] Vanclay JK (2007). On the robustness of the h-index. J Am Soc Inform Sci Technol.

[48] Kelly CD, Jennions MD (2006). The h index and career assessment by numbers. Trends Ecol Evol.

